# Supplementary material for: Effects of atherogenic diet supplemented with fermentable carbohydrates on metabolic responses and plaque formation in coronary arteries using a Saddleback pig model
Source: PLoS One. 2022 Oct 7;17(10):e0275214. doi: 10.1371/journal.pone.0275214 (PMC9543622; doi:10.1371/journal.pone.0275214)
Supplement: S1 File — (DOCX) [file pone.0275214.s001.docx]

**S1: ARRIVE guidelines 2.0 Checklist**

| Study design | 48 pig divided in 5 groups:   - 1 Baseline Groups (BL, n = 8): BL was slaughtered after adaption period (3 weeks) to obtain baseline values of coronary artery samples. All groups received the conventional diet during adaption period. - 4 Feeding groups (feeding period of 15 weeks)   - Atherogenic diet (AD, n = 10)   - Atherogenic diet + 5 % pectin (ADp, n = 10)   - Atherogenic diet + 5 % inulin (ADi, n = 10)   - Conventional diet (CD, n = 10) = control group - At the end of the feeding period pigs were slaughtered for sample collection   - Stunning: electrical stunning equipment (TGB 200; Hubert Haas, Neuler, Germany)   - Blood withdrawal: severing brachiocephalic trunk and jugular vein   - Pigs were slaughtered in accordance with European and German law [Council Regulation (EC) No 1099/2009 of 24 September 2009, Tierschutz-Schlachtverordnung, § 4 Tierschutzgesetz]. |
| --- | --- |
| Sample size | A total number of 48 pigs were included in this study. Pigs were divided into 5 groups:   - BL (n = 8) - AD (n = 10) - ADp (n = 10) - ADi (n = 10) - CD (n = 10)   Sample size calculation:   - Power analysis (power > 80 %, significance level at 5 %, SPSS Statistics 27.0, IBM, New York, USA) with the main target variable acetate as a function of carbohydrate intake (inulin, pectin) compared to the control group. - Published data in pigs assume differences of acetate in the chyme of 200mmol/kg dry mass in the chyme between the control group and the inulin / pectin fed animals. The standard variations varied between 50-62 mmol/kg dry mass in the chyme. The calculation of the required number of animals was between 8 and 9 pigs per group. We increase the number of animals to 10 per group to compensate for unexpected animal losses. |
| Inclusion and exclusion criteria | Including criteria:   - Breed: Saddle back pigs (5 litters, same sire) - Age: 5 months - Health status: Healthy (examined by clinical examination and blood check)   Excluding criteria:   - A score sheet was developed to establish the criteria for excluding animals from the study. The score was developed specifically for pigs and modified based on the results for pain assessment in pigs by Ison et al. (2016). - Scored parameter: general behaviour, condition score, feed and water intake, quality of faeces, breathing rate, body temperature, limbs, injuries (especially tail, ears and flanks), umbilical hernia, symptoms of gastric ulcers. - Score > 0 to 1 is reached for one parameter 🡪 animals were intensively observed (clinical examination three times a day) - Score > 1 is reached for more than one parameter or a score of 3 is reached for one parameter 🡪 the animals are excluded of the study and undergo further diagnostic examinations (e.g. further lameness diagnostics) and, if necessary, lege artis medical treatment (e.g. pain therapy or antibiosis). - If a complete recovery of the animals is possible, they are included in the trial again. - If individual animals cannot be recovered and included in the trial, these animals are euthanised to avoid further pain and suffering. |
| Randomisation | The pigs were randomly divided into five groups after weaning by the stable staff. These groups were maintained in the study to avoid rank fights between the animals. However, care was taken to ensure that animals from all litters and of all sexes were present in each group. |
| Blinding | The study was not blinded. |
| Outcome  measures | Outcome measures   - Feed intake - Body weight (BW) development - Body condition score (BCS) development - Back fat thickness (BFT) development - Serum parameters related to liver and fat metabolism: triglyceride (TG), bile acids (BA), hepatic triglyceride lipase (LIPC), cholesterol (CHOL), alkaline phosphatase (ALP), glutamate dehydrogenase (GLDH), aspartate aminotransferase (AST), gamma-glutamyl transferase (GGT) lactate dehydrogenase (LDH) and amylase (AMYL) - Serum metabolome - Fat content in faeces - Plaque formation in coronary arteries: plaque frequency per group and plaque size   The main target variable to determine the sample size was acetate in chyme as a function of carbohydrate intake (inulin, pectin) compared to the control group. The analysis of the short-chain fatty acids in chyme are not completed yet and will be published at a later date. |
| Statistical methods | Check for normal distribution:   - Shapiro-Wilks Test (SPSS Statistics 27.0, IBM, New York, USA)   Check for variance homogeneity:   - Levene Test (SPSS Statistics 27.0, IBM, New York, USA)   Normally distributed and homogenous data sets:   - BW, BCS, BFT (SPSS Statistics 27.0, IBM, New York, USA) CHOL and GGT (Statistica 14.0, TIBCO, Palo Alto, USA) - Repeated measures ANOVAs - Post hoc test: Turkey HSD test   Not normally distributed data sets:   - TG, BA, LIPC, AST, LDH, AMYL, ALP and plaque size (SPSS Statistics 27.0, IBM, New York, USA) - Kruskal-Wallis test with Bonferroni correction   Chi Square Test to evaluate plaque frequency per group (SPSS Statistics 27.0, IBM, New York, USA).  Principal component analysis (PCA) and Partial least Square-Discriminant Analysis (PLS-DA):   - NMR data of metabolomics (R version 4.0.2 2021, R Core Team) |
| Experimental animals | - 48 healthy, purebred saddleback pigs owned by the Institute of Animal Nutrition, Nutrition Diseases and Dietetics, Leipzig University - female (n = 21) and castrated male pigs (n = 27) from five litters with the same sire - pigs aged 5 months - mean (± SD) body weight: 97.5 ± 9.36 kg. - median and [25th / 75th] percentiles of body condition score (BCS): 3.13 [3.0 / 3.5] out of five - mean (± SD) back-fat thickness (BFT): 20.3 ± 2.08 cm - Pigs were housed in the same stable and separated in one pen per group according to the allotted treatment. The ambient temperature was 16–18 °C, and the humidity was 60–75 %. The pigs were bedded with wood shavings. - Water was provided ad libitum by using an automatic watering system. - Animals were adapted to the general experimental environment for at least 3 weeks. During the adaptation period, pigs were fed the same conventional diet. - The project was approved by the Ethics Committee for Animal Rights Protection of the Leipzig District Government (no. TVV 04/20) in accordance with German legislation for animal rights and welfare. - Two animal keepers provided the animals with food and cleaned the stable on a daily basis. - Health status of the animals was examined by a Veterinarian in two-day intervals by clinical examination, including evaluations of general behavior, feed and water intake, fecal quality, breathing rate, and body temperature. In addition, blood tests (blood count and chemistry) were performed before and after the feeding period. - Temperature and humidity in the stable were recorded daily. |
| Experimental procedures | Morphometric measurements  Body weight was measured weekly with a portable electronic scaling system (Minipond 21, Baumann Waagen und Maschinenbau GmbH, Thiersheim, Germany). BCS was evaluated weekly using a scale from 0 to 5 [Young et al. 2001]. Monthly, BFT was obtained by transcutaneous ultrasound measurements (Portable Ultrasonic Diagnostic System A6V, SonoScape Co., Shenzhen, China) of six measuring points according to the ABC-6-methode [Spanlang 2011].  Blood sampling  Blood samples were collected at the beginning of the study (t0; October, 8 to October,13, 2020). Blood was taken by single puncture of the left or right jugular vein. Follow up blood samples of the four feeding groups were sampled after one (t1; November, 11, 2020), two (t2; December, 17, 2020) and three months (t3; January, 21, 2021) of feeding the experimental diets. For blood chemistry and metabolome analyses serum tubes (Monovette^®^ Z, Sarstedt AG & Co. KG, Nümbrecht, Germany) containing coagulation activator were used. For blood count tubes containing EDTA (Monovette^®^ K3E (1.6 mg EDTA/mL), Sarstedt AG & Co. KG) were taken and immediately analysed after sampling. Serum tubes were centrifuged after 30 min of clotting at room temperature and then frozen in multiple aliquots of 1mL at −80°C until analysis.  Faeces sampling  Faecal samples were collected at the same time points (t0; t1; t2; t3) as the blood samples. Rectal faeces were collected from each animal. Pooled faeces samples were prepared for each feeding group and analysed directly.  Slaughtering process  Pigs were adapted and familiar with manual handling. All animals underwent a veterinary examination immediately before slaughtering and all pigs were diagnosed to be clinically healthy. Slaughter of the pigs took place on the same farm where the pigs were kept during the experiment (distance from the stable to the slaughterhouse: 200 m), which excluded long transportation stress. The pigs were individually stunned with an electric stunning system according to European and German law [Council Regulation (EC) No 1099/2009 of 24 September 2009, Tierschutz-Schlachtverordnung, § 4 Tierschutzgesetz] (TGB 200; Hubert Haas, Neuler, Germany, brain and brain-heart perfusion, minimum current of 1.3 A within the 1^st^ second, 250 V, alternating current with 50−100 Hz, perfusion duration with 1.3 A for at least 4 seconds, data were recorded by the stunning system) by the qualified butcher and then sacrificed by blood withdrawal within 10 seconds (knife with a blade length of at least 12 cm, stabbing direction in the jugular fossa 2−3 cm in front of the sternal apex in the direction of the opposite scapula, cut length of 2−3 cm, opening of the brachiocephalic trunk and jugular vein, blood loss of at least 3−4 L in 30 seconds). Thereafter, the carcasses were examined by an official veterinarian and declared edible. The carcasses were processed by regional butchers.  Sampling of coronary arteries  After slaughtering, hearts were separated in toto from the carcass for subsequent sampling and washed with isotonic saline (NaCl, Carl Roth GmbH + Co. KG, Karlsruhe, Germany). The left anterior descending branch of the left coronary artery (LAD) was manually flushed with isotonic saline and four 2-3 mm segments were removed. Two LAD segments per animal were shock-frozen in liquid nitrogen and then stored at -80 °C for the preparation of frozen sections. Two segments were fixed in 10 % neutral-buffered formalin (Sigma-Aldrich, St. Louis, USA) for at least two days for the preparation of polyethylene glycol-sections. |
| Results | 1. **Increases in BW, BCS, and BFT in the four feeding groups during the observation period.**  \| **Parameter in (%)** \| **Group AD** \| **Group ADp** \| **Group ADi** \| **Group CD** \| **P values** \| \| --- \| --- \| --- \| --- \| --- \| --- \| \| **BW** \| 61.2 ± 16.3 \| 58.1 ± 12.0 \| 62.1 ± 11.8 \| 58.9 ± 8.60 \| 0.82 \| \| **BSC** \| 32.1 ± 13.9 \| 33.8 ± 6.30 \| 33.6 ± 11.2 \| 33.0 ± 12.3 \| 0.99 \| \| **BFT** \| 110 ± 41.9 \| 90.9 ± 45.3 \| 109 ± 53.1 \| 82.3 ± 44.7 \| 0.42 \|   Data are presented as mean ± SD. Significant differences between feeding groups (n = 10) were identified by P values ≤ 0.05, using repeated measures ANOVA with Tukey’s HSD. AD, group fed atherogenic diet; ADp, group fed atherogenic diet + pectin; ADi, group fed atherogenic diet + inulin; CD, group fed conventional diet; BW, body weight; BCS, body condition score; BFT, back fat thickness. The observation period was 15 weeks.   1. **Crude lipid content in pooled fecal samples of each dietary group (AD, ADp, ADi, CD) at each sampling point (t0–t3).**  \| **CL in DM (%)** \| **Groups** \| **t0**  **(start)** \| **t1**  **(1 month)** \| **t2**  **(2 months)** \| **t3**  **(3 months)** \| \| --- \| --- \| --- \| --- \| --- \| --- \| \| **AD** \| 6.27 \| 24.6 \| 17.5 \| 21.1 \| \| **ADp** \| 6.44 \| 24.2 \| 25.3 \| 17.9 \| \| **ADi** \| 7.21 \| 21.8 \| 18.5 \| 20.9 \| \| **CD** \| 5.98 \| 4.10 \| 4.81 \| 4.14 \|   Data are presented as percentages (in DM) for one pool of ten animals per group. CL, crude lipid; DM, dry matter; AD, group fed an atherogenic diet (n = 10); ADp, group fed an atherogenic diet + pectin (n = 10); ADi, group fed an atherogenic diet + inulin (n = 10); CD, group fed a conventional diet (n = 10).   1. **Triglycerides, hepatic triglyceride lipase, bile acids, and cholesterol concentrations in serum of all groups at each sampling point (t0–t3).**  \| **Serum parameters** \| **Groups** \| **t0** \| **t1** \| **t2** \| **t3** \| \| --- \| --- \| --- \| --- \| --- \| --- \| \| **TG**  **(mmol/L)** \| **BL** \| 0.38  [0.28 / 0.49] \| n/a \| n/a \| n/a \| \| **AD** \| 0.34^▲^  [0.33 / 0.44] \| 0.73^#, a^  [0.62 / 0.82] \| 0.66^#, a^  [0.52 / 1.21] \| 0.40^▲^  [0.29 / 0.53] \| \| **ADp** \| 0.39^▲^  [0.28 / 0.50] \| 0.76^#, a^  [0.62 / 0.95] \| 0.62^▲#, ab^  [0.38 / 0.71] \| 0.43^▲^  [0.27 / 0.60] \| \| **ADi** \| 0.32^▲^  [0.30 / 0.41] \| 0.63^#■, a^  [0.44 / 0.82] \| 0.76^■, a^  [0.54 / 1.17] \| 0.43^▲#^  [0.26 / 0.63] \| \| **CD** \| 0.30  [0.25 / 0.34] \| 0.29^b^  [0.25 / 0.30] \| 0.31^b^  [0.24 / 0.35] \| 0.29  [0.25 / 0.32] \| \| **BA**  **(µmol/L)** \| **BL** \| 5.25  [3.83 / 5.70] \| n/a \| n/a \| n/a \| \| **AD** \| 4.70^▲^  [3.68 / 6.40] \| 21.2^#, a^  [17.2 / 23.5] \| 15.8^#, a^  [11.0 / 27.9] \| 11.3^▲#^  [7.73 / 17.6] \| \| **ADp** \| 4.85^▲^  [3.88 / 5.70] \| 17.4^#, a^  [14.0/ 21.2] \| 8.55^▲#, ab^  [7.08 / 15.3] \| 11.8^#^  [7.60 / 16.4] \| \| **ADi** \| 5.95^▲^  [4.25 / 11.8] \| 14.7^#, ab^  [7.48 / 22.3] \| 13.0^▲#, a^  [11.7 / 15.0] \| 12.6^▲#^  [6.30 / 13.5] \| \| **CD** \| 5.85  [5.00 / 8.30] \| 7.70^b^  [6.38 / 11.1] \| 6.55^b^  [4.38 / 10.5] \| 8.65  [6.25 / 11.3] \| \| **LIPC**  **(mmol/L)** \| **BL** \| 5.00  [4.25 / 5.00] \| n/a \| n/a \| n/a \| \| **AD** \| 4.00^▲^  [4.00 / 4.00] \| 6.50^#, a^  [6.00 / 7.00] \| 6.50^#, ab^  [5.00 / 7.50] \| 5.00^▲#^  [5.00 / 5.25] \| \| **ADp** \| 4.00^▲^  [4.00 / 4.00] \| 6.00^#■, a^  [6.00 / 7.25] \| 7.00^■, a^  [6.00 / 7.25] \| 5.00^▲#^  [5.00 / 5.00] \| \| **ADi** \| 4.00^▲^  [4.00 / 4.00] \| 6.00^#, a^  [5.75 / 6.25] \| 6.00^#, ab^  [5.00 / 6.25] \| 4.50^▲^  [4.00 / 5.00] \| \| **CD** \| 4.00^▲^  [4.00 / 4.25] \| 5.00^#, b^  [5.00 / 5.00] \| 6.00^#, b^  [5.00 / 6.00] \| 5.00^▲#^  [4.00 / 5.25] \| \| **CHOL**  **(mmol/L)** \| **BL** \| 2.37  [2.18 / 2.58] \| n/a \| n/a \| n/a \| \| **AD** \| 2.47  [2.27 / 2.66] \| 2.47^a^  [2.33 / 2.53] \| 2.32  [2.10 / 2.49] \| 2.49^a^  [2.17 / 2.71] \| \| **ADp** \| 2.24^▲#^  [2.04 / 2.31] \| 2.40^▲, ab^  [2.21 / 2.51] \| 2.43^▲^  [2.34 / 2.51] \| 2.14^#, ab^  [1.96 / 2.35] \| \| **ADi** \| 2.17^▲^  [1.98 / 2.35] \| 2.24^▲#, ab^  [2.14 / 2.37] \| 2.35^▲#^  [2.21 / 2.52] \| 2.38^#, a^  [2.24 / 2.48] \| \| **CD** \| 2.27^▲^  [2.09 / 2.36] \| 2.10^▲#, b^  [1.87 / 2.33] \| 2.14^▲#^  [1.92 / 2.29] \| 1.88^#, b^  [1.75 / 2.09] \|   Data are presented as medians and [25th / 75th] percentiles. ^▲#■^Different symbols indicate significant differences within a row (time-point differences). ^ab^Lowercase letters indicate significant effects within a column (group differences). Significant differences between the groups (BL, n = 8; AD, ADp, ADi, CD, n = 10) are identified by P values ≤ 0.05 using Kruskal–Wallis test with Bonferroni correction for TG, BA and repeated measures ANOVA with Tukey HSD for CHOL. BL, baseline group; AD, group fed atherogenic diet; ADp, group fed atherogenic diet + pectin; ADi, group fed atherogenic diet + inulin; CD, group fed conventional diet; TG, triglycerides; LIPC, hepatic triglyceride lipase; BA, bile acids; CHOL, cholesterol; n/a, not available.   1. **Parameters of LAD plaque formation in all groups.**  \| **Group** \| **Percentage of pigs within a group showing vascular lesions in LAD (%)** \| **Plaque size in plaque-positive pigs (µm², P = 0.33)** \| \| --- \| --- \| --- \| \| **BL** \| 63^ab^ \| 35061  [22930 / 71273] \| \| **AD** \| 50^a^ \| 157062  [73901 / 198848] \| \| **ADp** \| 100^b^ \| 40207  [21287 / 181180] \| \| **ADi** \| 70^ab^ \| 128539  [34196 / 212237] \| \| **CD** \| 70^ab^ \| 46162  [14275 / 139486] \|   Data show the plaque frequency per group (%) and plaque size (µm²) of the plaque-positive pigs per group, expressed as medians and [25th / 75th] percentiles. ^ab^Lowercase letters indicate significant effects within the columns. Significant differences between the groups (BL, n = 8; AD, ADp, ADi, CD, n = 10) were identified using the chi-squared test (P ≤ 0.05). LAD, left anterior descending branch of the left coronary artery; BL, baseline group; AD, group fed atherogenic diet; ADp, group fed atherogenic diet + pectin; ADi, group fed atherogenic diet + inulin; CD, group fed conventional diet. |
