# Supplementary material for: Effects of atherogenic diet supplemented with fermentable carbohydrates on metabolic responses and plaque formation in coronary arteries using a Saddleback pig model
Source: PLoS One. 2022 Oct 7;17(10):e0275214. doi: 10.1371/journal.pone.0275214 (PMC9543622; doi:10.1371/journal.pone.0275214)
Supplement: S2 Table — (DOCX) [file pone.0275214.s003.docx]

**S3: Serum parameters of liver and lipid metabolism of all groups at each sampling point (t0–t3).**

| **Serum parameters** | **Groups** | **t0** | **t1** | **t2** | **t3** |
| --- | --- | --- | --- | --- | --- |
| **TG**  **(mmol/L)** | **BL** | 0.38  [0.28 / 0.49] | n/a | n/a | n/a |
|  | **AD** | 0.34^▲^  [0.33 / 0.44] | 0.73^#, a^  [0.62 / 0.82] | 0.66^#, a^  [0.52 / 1.21] | 0.40^▲^  [0.29 / 0.53] |
|  | **ADp** | 0.39^▲^  [0.28 / 0.50] | 0.76^#, a^  [0.62 / 0.95] | 0.62^▲#, ab^  [0.38 / 0.71] | 0.43^▲^  [0.27 / 0.60] |
|  | **ADi** | 0.32^▲^  [0.30 / 0.41] | 0.63^#■, a^  [0.44 / 0.82] | 0.76^■, a^  [0.54 / 1.17] | 0.43^▲#^  [0.26 / 0.63] |
|  | **CD** | 0.30  [0.25 / 0.34] | 0.29^b^  [0.25 / 0.30] | 0.31^b^  [0.24 / 0.35] | 0.29  [0.25 / 0.32] |
| **LIPC**  **(mmol/L)** | **BL** | 5.00  [4.25 / 5.00] | n/a | n/a | n/a |
|  | **AD** | 4.00^▲^  [4.00 / 4.00] | 6.50^#, a^  [6.00 / 7.00] | 6.50^#, a^  [5.00 / 7.50] | 5.00^▲^  [5.00 / 5.25] |
|  | **ADp** | 4.00^▲^  [4.00 / 4.00] | 6.00^#, a^  [6.00 / 7.25] | 7.00^#, a^  [6.00 / 7.25] | 5.00^▲^  [5.00 / 5.00] |
|  | **ADi** | 4.00^▲^  [4.00 / 4.00] | 6.00^#, a^  [5.75 / 6.25] | 6.00^#, a^  [5.00 / 6.25] | 4.50^▲^  [4.00 / 5.00] |
|  | **CD** | 4.00^▲^  [4.00 / 4.25] | 5.00^#, a^  [5.00 / 5.00] | 6.00^#, a^  [5.00 / 6.00] | 5.00^▲^  [4.00 / 5.25] |
| **BA**  **(µmol/L)** | **BL** | 5.25  [3.83 / 5.70] | n/a | n/a | n/a |
|  | **AD** | 4.70^▲^  [3.68 / 6.40] | 21.2^#, a^  [17.2 / 23.5] | 15.8^#, a^  [11.0 / 27.9] | 11.3^▲#^  [7.73 / 17.6] |
|  | **ADp** | 4.85^▲^  [3.88 / 5.70] | 17.4^#, a^  [14.0/ 21.2] | 8.55^▲#, ab^  [7.08 / 15.3] | 11.8^#^  [7.60 / 16.4] |
|  | **ADi** | 5.95^▲^  [4.25 / 11.8] | 14.7^#, ab^  [7.48 / 22.3] | 13.0^▲#, a^  [11.7 / 15.0] | 12.6^▲#^  [6.30 / 13.5] |
|  | **CD** | 5.85  [5.00 / 8.30] | 7.70^b^  [6.38 / 11.1] | 6.55^b^  [4.38 / 10.5] | 8.65  [6.25 / 11.3] |
| **CHOL**  **(mmol/L)** | **BL** | 2.37  [2.18 / 2.58] | n/a | n/a | n/a |
|  | **AD** | 2.47  [2.27 / 2.66] | 2.47^a^  [2.33 / 2.53] | 2.32  [2.10 / 2.49] | 2.49^a^  [2.17 / 2.71] |
|  | **ADp** | 2.24^▲#^  [2.04 / 2.31] | 2.40^▲, ab^  [2.21 / 2.51] | 2.43^▲^  [2.34 / 2.51] | 2.14^#, ab^  [1.96 / 2.35] |
|  | **ADi** | 2.17^▲^  [1.98 / 2.35] | 2.24^▲#, ab^  [2.14 / 2.37] | 2.35^▲#^  [2.21 / 2.52] | 2.38^#, a^  [2.24 / 2.48] |
|  | **CD** | 2.27^▲^  [2.09 / 2.36] | 2.10^▲#, b^  [1.87 / 2.33] | 2.14^▲#^  [1.92 / 2.29] | 1.88^#, b^  [1.75 / 2.09] |
| **ALP**  **(U/L)** | **BL** | 134  [107 / 152] | n/a | n/a | n/a |
|  | **AD** | 116  [92.5 / 213] | 142^a^  [121 / 245] | 133  [79.5 / 216] | 127^a^  [88.0 / 238] |
|  | **ADp** | 182  [142 / 224] | 162^a^  [137 / 211] | 156  [129 / 190] | 111^a^  [101 / 158] |
|  | **ADi** | 170  [123 / 291] | 144^a^  [115 / 228] | 162  [134 / 285] | 162^a^  [113 / 269] |
|  | **CD** | 128  [95.8 / 152] | 144^a^  [106 / 167] | 146  [115 / 167] | 134^a^  [120 / 153] |
| **AST**  **(U/L)** | **BL** | 29.1^ac^  [28.5 / 34.0] | n/a | n/a | n/a |
|  | **AD** | 36.6^▲, b^  [34.1 / 38.9] | 29.6^#^  [23.8 / 33.3] | 28.8^▲#^  [27.9 / 35.4] | 26.8^#^  [24.6 / 30.1] |
|  | **ADp** | 37.2^▲, ab^  [31.8 / 41.8] | 25.1^#^  [22.6 / 31.9] | 29.6^▲#^  [26.0 / 35.3] | 28.3^▲#^  [24.4 / 40.2] |
|  | **ADi** | 32.2^▲, abc^  [29.0 / 36.1] | 23.4^#^  [20.3 / 26.2] | 26.0^▲#^  [23.4 / 34.0] | 24.2^#^  [22.7 / 26.1] |
|  | **CD** | 29.1^c^  [26.9 / 31.4] | 27.0  [24.6 / 31.5] | 29.3  [25.0 / 34.5] | 28.3  [25.7 / 32.3] |
| **GGT**  **(U/L)** | **BL** | 34.6  [26.8 / 45.9] | n/a | n/a | n/a |
|  | **AD** | 32.6  [29.9 / 35.6] | 32.0  [25.6 / 34.3] | 33.1  [26.6 / 35.4] | 32.7  [27.5 / 35.8] |
|  | **ADp** | 36.2  [31.6 / 46.6] | 36.7  [31.8 / 46.0] | 41.0  [37.2 / 52.8] | 35.6  [31.7 / 46.1] |
|  | **ADi** | 34.0  [20.2 / 37.6] | 31.7  [21.3 / 37.4] | 35.1  [23.2 / 39.0] | 33.5  [23.1 / 39.8] |
|  | **CD** | 30.2  [23.3 / 36.3] | 33.5  [25.3 / 46.5] | 33.4  [29.1 / 46.3] | 32.1  [23.5 / 44.4] |
| **LDH**  **(U/L)** | **BL** | 450  [432 / 482] | n/a | n/a | n/a |
|  | **AD** | 507  [437 / 532] | 504  [456 / 544] | 526  [469 / 604] | 497  [447 / 548] |
|  | **ADp** | 450^▲^  [425 / 489] | 531^▲#^  [482 / 556] | 585^#^  [542 / 636] | 544^#^  [503 / 602] |
|  | **ADi** | 462  [422 / 532] | 455  [426 / 476] | 537  [460 / 570] | 479  [454 / 546] |
|  | **CD** | 465^▲^  [408 / 490] | 500^▲#^  [470 / 564] | 557^#^  [468 / 718] | 500^▲#^  [465 / 541] |
| **AMYL**  **(U/L)** | **BL** | 2393  [2113 / 3307] | n/a | n/a | n/a |
|  | **AD** | 2424  [2393 / 2925] | 2277  [2025 / 2893] | 2339  [2248 / 2861] | 2399  [2263 / 2828] |
|  | **ADp** | 2913  [2325 / 3358] | 2795  [2090 / 3343] | 2988  [2103 / 3480] | 2868  [2036 / 3143] |
|  | **ADi** | 2762  [2242 / 3212] | 2482  [1980 / 2958] | 2766  [2160 / 3247] | 2774  [2311 / 3429] |
|  | **CD** | 2521  [1972 / 3100] | 2829  [2207 / 3318] | 2982  [2218 / 3615] | 2985  [2186 / 3573] |

Data are presented as medians and [25th / 75th] percentiles. ^▲#■^Different symbols indicate significant differences within a row (time-point differences). ^ab^Lowercase letters indicate significant effects within a column (group differences). Significant differences between the groups (BL, n = 8; AD, ADp, ADi, CD, n = 10) are identified by P values ≤ 0.05 using Kruskal–Wallis test with Bonferroni correction for TG, BA, LIPC, AST, LDH, AMYL, ALP and repeated measures ANOVA with Tukey HSD for CHOL and GGT. BL, baseline group; AD, group fed atherogenic diet; ADp, group fed atherogenic diet + pectin; ADi, group fed atherogenic diet + inulin; CD, group fed conventional diet; TG, triglycerides; LIPC, hepatic triglyceride lipase; BA, bile acids; CHOL, cholesterol; ALP, alkaline phosphatase; AST, aspartate aminotransferase; GGT, gamma-glutamyl transferase; LDH, lactate dehydrogenase; AMYL, amylase; n/a, not available.
